# The Development of a Nomogram Predictive Model for Intracardiac Thrombosis Risk: A Study Based on Risk Factors in Patients with Acute Myocardial Infarction

**DOI:** 10.3390/biomedicines13030679

**Published:** 2025-03-10

**Authors:** Xiaowei Huo, Zizhu Lian, Peizhu Dang, Yongjian Zhang

**Affiliations:** 1 Department of Cardiovascular Medicine, The First Affiliated Hospital of Xi’an Jiao Tong University, Xi’an 710061, China; hxwamy0916@xjtu.edu.cn; 2 Department of Cardiovascular Surgery, The First Affiliated Hospital of Xi’an Jiao Tong University, Xi’an 710061, China; lisalzz2651092799@163.com

**Keywords:** acute myocardial infarction, intracardiac thrombosis, risk factors, nomogram predictive model

## Abstract

**Background/Objectives**: Intracardiac thrombosis (ICT) is a serious complication in acute myocardial infarction (AMI) patients. This study aimed to identify potential risk factors of ICT in AMI patients, providing valuable insights for clinical management. **Methods**: A case–control study was conducted involving consecutive AMI patients admitted to the First Affiliated Hospital of Xi’an Jiaotong University between January 2019 and December 2022. Binary logistic regression identified independent risk factors of ICT and a nomogram prediction model was constructed and validated for accuracy. **Conclusions**: A total of 7341 patients with ICT and 74 without ICT were included. Multivariate logistic regression identified male gender, acute anterior wall myocardial infarction (AWMI), ventricular aneurysm, and lower prothrombin activity as independent risk factors of ICT in AMI patients. A nomogram based on these factors demonstrated excellent performance (AUC: 0.910, 95% CI: 0.877–0.943, *p* < 0.001), with calibration and sensitivity analyses confirming its robustness. This nomogram provides an accurate tool for predicting ICT risk, facilitating personalized management and early intervention in AMI patients.

## 1. Introduction

Acute myocardial infarction (AMI) refers to myocardial tissue death resulting from an unstable ischemic condition [1]. The prognosis of AMI is closely related to its complications. Intracardiac thrombosis (ICT) is a rare but severe complication in AMI patients, involving pathological thrombus formation within the heart chambers, including the left atrial appendage [2,3]. Currently, no global reports exist on the incidence of ICT in AMI patients. A large-scale analysis of 11,724 autopsy reports found that 276 (2.4%) had ICT, with most deaths (73.3%) attributed to systemic embolism or pulmonary embolism [4]. Regarding intracardiac thrombi, research mainly focuses on the left ventricular thrombus (LVT) in AMI patients, with an incidence of about 4% [5,6]. The onset of ICT is often subtle, making early diagnosis challenging, yet it is associated with high mortality and serious complications. Detachment of the thrombus can lead to pulmonary embolism, cerebral infarction, and other events. Furthermore, the thrombus can impair cardiac function, resulting in heart failure or even cardiac rupture [7]. Therefore, early diagnosis of ICT in AMI patients is critical.

Advancements in diagnostic technologies have increased the detection rate of ICT among AMI patients [8]. However, most studies focus on symptomatic embolic events, which are typically diagnosed after other severe complications have occurred, potentially underestimating the true incidence of ICT. Transthoracic echocardiography (TTE) is the most used diagnostic tool, but its accuracy is limited by operator variability [9]. Delayed-enhancement cardiac magnetic resonance imaging (DE-CMR) is considered the gold standard in ICT diagnosis but its high cost limits its routine clinical use [10]. Given the limitations of current diagnostic methods, early screening and management of high-risk ICT patients remain complex and urgent issues.

Currently, most studies on this topic focus on exploring the risk factors of LVT after AMI [11]; there are no clinical studies that specifically examine the risk factors of ICT in AMI patients. Therefore, identifying specific risk factors of ICT in AMI patients and developing reliable predictive models are of paramount importance. Our study aims to analyze hospital examination results of AMI patients and develop a nomogram-based prediction model for ICT.

## 2. Materials and Methods

### 2.1. Study Design and Patients

This retrospective observational study was carried out at the First Affiliated Hospital of Xi’an Jiao Tong University (No. 277, West Yanta Road, Xi’an, Shaanxi Province, 710061, China), focusing on consecutive patients diagnosed with AMI between January 2019 and December 2022. Diagnoses of AMI and ICT conformed to universal criteria [12,13] and treatment protocols adhered to the 2023 ESC guidelines for the management of acute coronary syndromes [14]. The study included patients with confirmed AMI cases, covering both non-ST-segment elevation myocardial infarction (NSTEMI) and ST-segment elevation myocardial infarction (STEMI). In patients with ICT, diagnosis required the detection of thrombi at various cardiac locations via echocardiography during hospitalization [3,15]. These locations included the left and right ventricles, left and right atria, atrial appendages, apex, and multiple other sites. We excluded patients with missing laboratory data for most of the tests. For patients with missing values in only a few laboratory items, we applied multiple imputation to handle the missing data.

Patients were grouped into those with ICT and those without ICT. We then compared demographic details, laboratory results, and other clinical factors between the two groups to identify risk factors linked to ICT.

**Figure 1:**
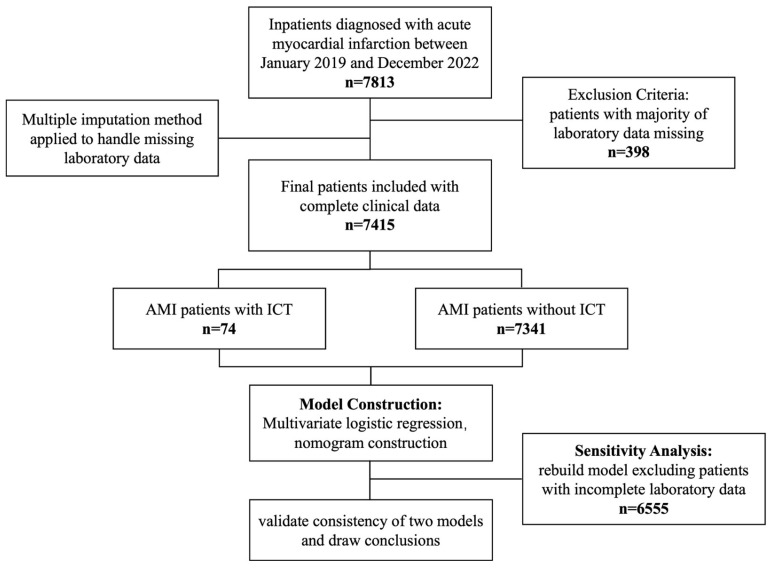
A flowchart of the study.

This study was conducted in accordance with the Declaration of Helsinki (1975, revised in 2013) and received prior ethical approval from the Institutional Review Board of Xi’an Jiao Tong University (Approval No. XJTU1AF2025LSYY-359). As a retrospective analysis utilizing fully anonymized clinical data obtained from the Biobank of The First Affiliated Hospital of Xi’an Jiao Tong University, the requirement for written informed consent was formally waived by the ethics committee. All data-handling procedures complied with the biobank’s established protocols for the secondary use of biomedical data. The study flowchart was presented below (Figure 1).

### 2.2. Data Collection

Comprehensive baseline data were meticulously collected upon admission, including demographic details, specific AMI locations ascertained from initial electrocardiograms (ECG) [12,14], existing comorbidities, and Killip classification for heart failure. Laboratory data were obtained from the hospital’s electronic health records and included a range of tests, such as complete blood counts, comprehensive metabolic panels, renal function markers (e.g., blood urea nitrogen and serum creatinine), cardiac biomarkers like NT-proBNP, liver function assessments, D-dimer levels, and additional coagulation-related parameters. All laboratory measurements were obtained immediately following admission to ensure their accuracy and consistency.

### 2.3. Statistical Analyses

We initiated our analysis by evaluating continuous variables for normality, presenting them as either means ± standard deviations (SDs) or as medians with interquartile ranges (IQRs), depending on their distribution pattern. Categorical variables were detailed using frequencies and percentages. We employed independent samples *t*-tests for the analysis of normally distributed continuous variables during group comparisons. Conversely, the Mann–Whitney U test was utilized for variables with a non-normal distribution. Comparisons of categorical data were made using Pearson’s chi-square test or Fisher’s exact test where necessary.

Our exploratory phase involved univariate logistic regression to pinpoint potential risk factors of ICT in AMI patients. Significant variables (*p* < 0.05) were then incorporated into a multivariate logistic regression to ascertain independent predictors of ICT. The strength of these associations was expressed through odds ratios (ORs) and 95% confidence intervals (CIs). Based on insights gained from the multivariate analysis, we developed a clinical nomogram to predict the individual risk of ICT in AMI patients. We evaluated the model’s efficacy using the area under the receiver operating characteristic (ROC) curve (AUC). To assess model calibration, we used calibration curves that compared predicted probabilities of ICT with observed outcomes, where the *x*-axis represents the estimated risk of ICT and the *y*-axis displays the observed likelihood of its occurrence. The ideal prediction is depicted by a diagonal dashed line while the pre- and post-adjustment calibrations are shown by dashed and solid lines, respectively.

To ensure the robustness and reliability of our findings, we conducted a thorough sensitivity analysis, excluding any cases with incomplete data to avoid bias and improve the generalizability of our results. This refined dataset was subsequently reanalyzed to confirm the predictive accuracy and performance metrics of our model.

Statistical analyses were performed using SPSS software (version 27.0, IBM, Armonk, NY, USA) and R software (version 4.4.2), applying two-tailed tests with a significance level maintained at *p* < 0.05.

## 3. Results

### 3.1. The Subsection of Characteristics of the Studied Population

A total of 7813 patients diagnosed with AMI were treated at the First Affiliated Hospital of Xi’an Jiao Tong University. After excluding 398 patients due to incomplete laboratory data, 7415 patients remained eligible for analysis. To address missing laboratory data, we performed five multiple imputations and combined the results using Rubin’s rule. This approach was chosen to minimize bias and maximize the use of available data, ensuring robust estimates of risk factors. Among the analyzed cohort, 74 patients (0.99%) were diagnosed with ICT. The clinical characteristics of the studied participants are summarized in Table 1. Our findings indicate that male patients were at a significantly greater risk of developing ICT following AMI. Moreover, individuals with acute anterior wall myocardial infarction (AWMI) or those presenting with a ventricular aneurysm were found to be at a markedly higher risk of ICT, with both conditions showing statistically significant associations (*p* < 0.001). In laboratory analyses, the ICT group exhibited elevated levels of D-dimer, prothrombin time (PT), and international normalized ratio (INR), while prothrombin activity was notably reduced. The observed differences in D-dimer, PT, INR, and prothrombin activity were all statistically significant (*p* < 0.001).

### 3.2. Risk Factors of ICT in AMI Patients

We assessed baseline demographic and clinical characteristics between the ICT and non-ICT groups, including multicollinearity testing of the variables involved. Variables with a variance inflation factor (VIF) exceeding 10, such as white blood cell count, lymphocyte count, and neutrophil count, were excluded from the analysis. Variables with significant differences (*p* < 0.05) and of clinical relevance were included in the univariate logistic regression analysis (Table 2). Univariate logistic regression identified potential risk factors, with statistical significance at a *p*-value ≤ 0.05, and advanced to multivariate logistic regression. This analysis confirmed several independent risk factors of ICT post-AMI: male gender (OR: 2.936; 95% CI: 1.304–6.609; *p* = 0.009), AWMI (OR: 5.186; 95% CI: 2.569–10.467; *p* < 0.001), ventricular aneurysm (OR: 21.216; 95% CI: 12.455–36.138; *p* < 0.001), and decreased prothrombin activity (OR: 1.039; 95% CI: 1.018–1.062; *p* < 0.001), as detailed in Table 3.

### 3.3. The Construction of the Nomogram Model

**Figure 2:**
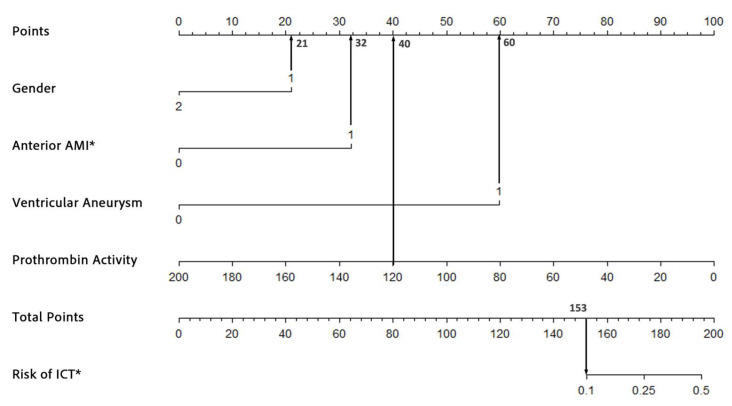
An example of the nomogram used to predict the risk of ICT formation in AMI patients. * ICT, intracardiac thrombosis; AMI, acute myocardial infarction.

The nomogram was developed based on the results of the multivariate logistic regression analysis. Four independent risk factors—male gender, acute anterior wall myocardial infarction (AWMI), ventricular aneurysm, and decreased prothrombin activity—were identified and included in the model. Variance inflation factors (VIFs) confirmed no multicollinearity among these variables (VIFs: 1.004, 1.359, 1.075, and 1.008). The regression coefficients for each predictor were scaled to assign points on the nomogram. For example, the coefficient for male gender was converted to a score of 21 points, while ventricular aneurysm, being the strongest predictor, was assigned 60 points. The total score for each patient was calculated by summing the points for all predictors and the corresponding risk of ICT was estimated using the nomogram’s probability scale. The nomogram provides clinicians with a user-friendly tool to estimate the risk of ICT in AMI patients. For instance, a 55-year-old male patient (21 points) presenting with AWMI (32 points), ventricular aneurysm (60 points), and a prothrombin activity of 120% (40 points) would receive a total score of 153 points, corresponding to a 10% risk of ICT (Figure 2). The nomogram is intended for use at the time of AMI diagnosis, leveraging data collected within the first 24 h of hospitalization; it is a practical tool for clinicians to assess individual risk and tailor thrombosis prevention strategies.

### 3.4. Model Accuracy Assessment

**Figure 3:**
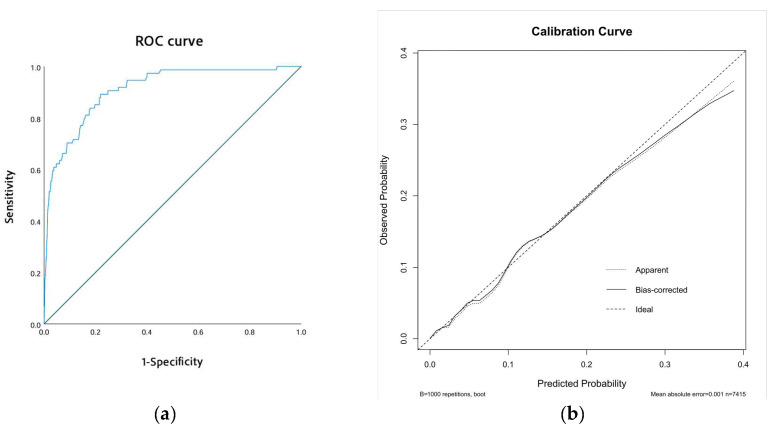
Model performance. (**a**) The ROC curve of the model. (**b**) The calibration curve of the model.

The performance of our predictive model was assessed through receiver operating characteristic (ROC) curves and calibration curves. The ROC curve revealed an area under the curve (AUC) of 0.910 (95% CI: 0.877–0.943, *p* < 0.001), indicating excellent discriminatory ability (Figure 3a). Additionally, the calibration curve showed strong alignment between predicted probabilities and observed outcomes. This was further supported by the Hosmer–Lemeshow test, which yielded a chi-square value of 6.974 and a *p*-value of 0.538, confirming the model’s reliable calibration (Figure 3b).

### 3.5. Sensitivity Analysis

**Figure 4:**
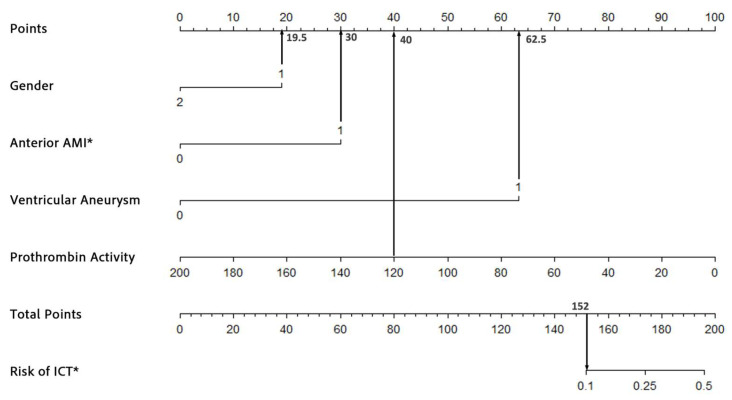
An example of the nomogram after excluding all missing data. * ICT, intracardiac thrombosis; AMI, acute myocardial infarction.

**Figure 5:**
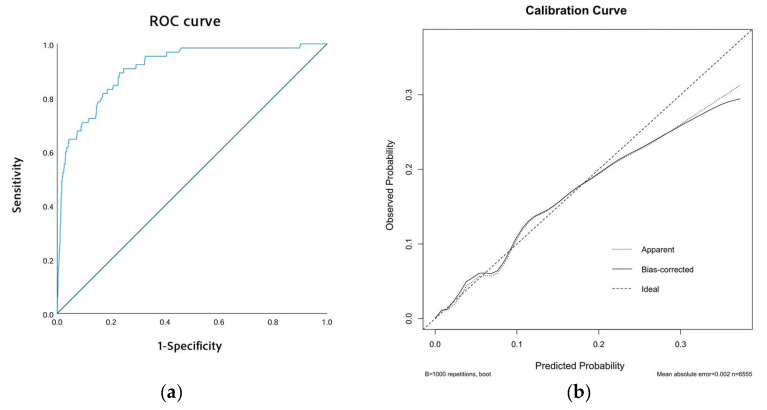
Model performance after excluding all missing data. (**a**) The ROC curve of the model. (**b**) The calibration curve of the model.

To assess the robustness and accuracy of the model, we conducted a sensitivity analysis. In the sensitivity analysis, we excluded patients with incomplete laboratory data, resulting in a final cohort of 6555 patients. We re-ran the logistic regression analysis and reconstructed the nomogram using the same patient characteristics as the original model. As shown in Table 3, the logistic regression analysis identified the same four significant risk factors as those obtained with the imputed dataset. For instance, a male patient (19.5 points) with AWMI (30 points), ventricular aneurysm (62.5 points), and a prothrombin activity of 120% (40 points) would have a cumulative score of 152, corresponding to an estimated risk of 10%. This demonstrated consistency in both the total score and ICT risk estimation (Figure 4), further supporting the robustness of the model. The ROC and calibration curves, as derived from the sensitivity analysis, are presented in Figure 5a,b, respectively. The ROC curve yielded an area under the curve (AUC) of 0.908 (95% CI: 0.872–0.945, *p* < 0.001), demonstrating robust discriminative ability. Furthermore, the calibration curve displayed excellent concordance between predicted probabilities and observed outcomes, which was substantiated by a Hosmer–Lemeshow test with a chi-square value of 7.334 and a *p*-value of 0.501. These findings underscore the model’s stability and reliability in predicting the risk of ICT in patients with AMI.

## 4. Discussion

We included 7415 patients diagnosed with AMI during hospitalization, of whom 74 developed ICT. The independent risk factors of ICT after AMI were identified as male gender, anterior wall infarction, presence of ventricular aneurysm, and lower prothrombin activity. Based on these four risk factors, we developed a robust nomogram prediction model.

Gender plays a pivotal role in the development of ICT. Our study demonstrates that male patients are at significantly higher risk of ICT compared to female patients, which aligns with findings from prior research. A meta-analysis shows that male patients with AMI experience a higher incidence of thromboembolic complications, including ICT, with an adjusted odds ratio (OR) of 1.6. This underscores the gender-related disparity in ICT incidence following AMI [16]. Several underlying mechanisms may contribute to this increased risk. First, the protective effects of estrogen are a critical factor. Estrogen enhances the activity of anticoagulant proteins, such as protein C, improves endothelial function, and inhibits platelet aggregation [17]. The absence of estrogen in males removes this cardiovascular protection, thereby increasing the risk of thrombosis [18]. Furthermore, males may exhibit less favorable endothelial function, rendering them more susceptible to endothelial injury, a key initiator of thrombosis [19,20]. Anatomically, male hearts are typically larger and have thicker ventricular walls, which lead to higher blood flow velocity and increased shear stress on the vascular endothelium. This heightened shear stress contributes to endothelial damage, thereby raising the likelihood of ICT formation [21,22]. Additionally, lifestyle factors, such as higher rates of smoking, alcohol consumption, and poor dietary habits among men, exacerbate these risks [18]. In conclusion, male gender is a robust predictor of ICT risk. This finding emphasizes the need for gender-specific risk assessments in clinical practice and highlights the importance of tailored prevention and treatment strategies to address gender-based disparity in ICT outcomes.

AWMI is a significant risk factor of ICT, consistent with previous studies. In a prospective study by Andre Keren et al., 31% of patients with AWMI developed thrombus prior to discharge, compared to none in the inferior wall MI group (*p* < 0.001) [23,24]. Involvement of the left anterior descending artery (LAD) in AWMI leads to extensive myocardial injury and necrosis of the anterior wall, impairing left ventricular function. This myocardial necrosis induces local wall motion abnormalities, contributing to blood stagnation and elevating the risk of thrombus formation [25]. Post-infarction structural remodeling triggers hemodynamic changes, creating low-flow regions near the infarct zone, which are high-risk areas for thrombosis [25,26]. Additionally, AWMI can cause ventricular electrophysiological changes, including atrial fibrillation (AF), which is closely associated with thrombus formation due to irregular atrial contractions and blood stasis [27]. This relationship underscores the strong association between AWMI and elevated ICT incidence, highlighting the importance of targeted monitoring. Implementing routine thrombus screening and proactive anticoagulation therapy in patients with AWMI could further reduce ICT incidence.

Ventricular aneurysm significantly differed between the ICT and control groups in our study. Andre et al. identified ventricular aneurysm as an independent risk factor of thrombus formation during hospitalization, with 13 out of 130 patients developing new thrombi over a six-month follow-up period [23]. Ventricular aneurysm, primarily caused by AWMI, leads to myocardial injury, scar formation, and ventricular wall bulging. This results in increased myocardial compliance, impaired contractile function, and blood stasis. Previous studies have linked local contractile dysfunction in the ventricular aneurysm region to ICT formation [28]. Reduced blood flow and disturbed hemodynamics in this area are key mechanisms for thrombus development [25]. Additionally, ventricular aneurysm alters ventricular structure, promoting thrombus retention through fibrosis and scarring, which provide surfaces for thrombus adhesion and growth [29]. Long-term anticoagulation is often required in AMI patients with ventricular aneurysm to manage chronic blood stasis, necessitating more aggressive anticoagulation to prevent thrombus recurrence. Optimizing anticoagulation therapy by adjusting dosages and duration based on individual patient risk profiles could enhance thrombus recurrence prevention in these patients [30].

Our analysis revealed a significant association between reduced prothrombin activity and ICT occurrence, highlighting the role of coagulation dysfunction in ICT pathogenesis. Previous studies have shown a correlation between abnormal coagulation markers and myocardial injury. For instance, a study of 987 STEMI patients found a significant correlation between peak TnT and D-dimer and F1 + 2 as well as between NT-proBNP and D-dimer and F1 + 2 [31,32]. Myocardial injury contributes to thrombus formation through decreased coagulation capacity, which weakens stable clot formation and promotes unstable clots prone to fragmentation and reformation, increasing ICT risk [33]. Additionally, lower prothrombin activity may exacerbate thrombus formation by modulating the inflammatory response, as thrombosis in AMI patients is closely associated with inflammation markers like C-reactive protein (CRP) and interleukins, which enhance thrombotic activity [34]. Targeting prothrombin activity through therapeutic interventions, such as prothrombin complex concentrates or novel anticoagulants, may be strategies to stabilize clot formation and reduce ICT incidence in AMI patients.

Compared to previous clinical research on similar topics, our study presents several significant advantages. First, we developed a nomogram integrating four key risk factors—male gender, AWMI, ventricular aneurysm, and reduced prothrombin activity—which consolidates multiple risk factors into a single, user-friendly model. This enables precise prediction of ICT risk through simple calculations, facilitating clinical decision-making and personalized medicine. Second, our large sample size enhances the model’s applicability across diverse patient populations. Third, multiple validation methods, including ROC curve analysis and calibration curves, confirm the model’s accuracy and robustness. Sensitivity analyses further support the model’s reliability, highlighting its potential utility in improving clinical outcomes. The nomogram is a practical tool for clinicians to identify high-risk patients and tailor management strategies accordingly. For patients with a high predicted risk of ICT (e.g., a nomogram score ≥ 150), we recommend a comprehensive approach including the following: 1. Imaging surveillance: regular transthoracic echocardiography (TTE) to monitor thrombus formation, particularly in patients with anterior wall infarction or ventricular aneurysm. 2. Prophylactic anticoagulation: the initiation of low-molecular-weight heparin (e.g., enoxaparin) in high-risk patients without contraindications. 3. Therapeutic anticoagulation: an escalation to therapeutic anticoagulation (e.g., warfarin or direct oral anticoagulants) if a thrombus is detected, following current guidelines [3]. This integrated approach can help reduce the incidence of ICT and improve outcomes in high-risk AMI patients.

This study has several limitations. First, although our sample size of 7415 AMI patients is substantial, it is confined to a single center, which may limit the generalizability of the findings. Second, the ICT incidence rate of 1% is lower than that reported in other studies (5–8%) [35,36]. This may be due to the lack of recent prevalence data as well as advancements in treatments that reduce ICT incidence. Moreover, as a leading medical center in Northwestern China, our center’s timely interventions have significantly lowered complication rates. Additionally, the retrospective design introduces potential selection and information biases. Future larger-scale multi-center studies are needed to validate these findings and address regional variations. Despite these limitations, this study is the first to investigate ICT risk factors in AMI patients, filling a significant gap in research.

## 5. Conclusions

In conclusion, this study identifies male gender, AWMI, ventricular aneurysm and lower prothrombin activity as significant risk factors of an increased incidence of ICT in patients with AMI. Given the association between these risk factors and ICT, clinicians should closely monitor AMI patients who present with these characteristics. Based on these risk factors, we have developed a nomogram model to facilitate clinical pre-assessment of ICT risk. Validation of the model demonstrated good accuracy, with the calibration curve showing strong concordance between predicted probabilities and actual outcomes, as confirmed by a Hosmer–Lemeshow test (chi-square = 6.974, *p* = 0.538), affirming the model’s reliable calibration. This model offers clinicians a visual tool to estimate the likelihood of ICT in AMI patients, utilizing four readily available clinical variables. By stratifying patients according to their risk profile, the nomogram serves as a powerful tool for early prevention and diagnosis of ICT, ultimately guiding more individualized management strategies for AMI patients. For patients identified as high-risk, adopting more aggressive treatment strategies is crucial to improving their prognosis.

## Figures and Tables

**Table 1:** Baseline characteristics of patients with AMI.

Variables	Total (n = 7415)	Control Group (n = 7341)	ICT Group (n = 74)	*p*-Value
Enrollment basic characteristics				
Age (years, IQR)	61.00 (53.0–69.0)	61.00 (53.00–69.00)	60.00 (49.25–69.75)	0.408
Female (n, %)	1407 (18.98)	1400 (19.07)	7 (9.46)	0.036
Location of AMI				
AWMI (n, %)	2185 (29.47)	2122 (28.91)	63 (85.14)	<0.001
Comorbidities				
Ventricular aneurysm (n, %)	280 (3.78)	237 (3.23)	43 (58.11)	<0.001
AF (n, %)	366 (4.94)	361 (4.92)	5 (6.76)	0.648
Hypertension (n, %)	3908 (52.70)	3870 (52.72)	38 (51.35)	0.815
Diabates (n, %)	2187 (29.49)	2163 (29.46)	24 (32.43)	0.577
CKD (n, %)	160 (2.16)	159 (2.17)	1 (1.35)	0.938
Hyperlipemia (n, %)	5345 (72.08)	5297 (72.16)	48 (64.86)	0.164
Low HDLC (n, %)	5052 (68.13)	5006 (68.19)	46 (62.16)	0.268
Killip classification (n, %)				
I	5848 (78.87)	5805 (79.08)	43 (58.11)	<0.001
II	1170 (15.78)	1144 (15.58)	26 (35.14)	<0.001
III	156 (2.10)	153 (2.08)	3 (4.05)	0.443
IV	241 (3.25)	239 (3.26)	2 (2.70)	1.000
Laboratory results				
WBC (×10^9^/L, IQR)	8.67 (6.76–11.16)	8.65 (6.76–11.15)	9.43 (7.34–11.64)	0.072
NEUs (×10^9^/L, IQR)	6.41 (4.66–9.00)	6.40 (4.65–8.99)	7.31 (5.31–9.69)	0.026
LYMs (×10^9^/L, IQR)	1.41 (1.03–1.88)	1.42 (1.03–1.88)	1.33 (0.94–1.71)	0.087
Creatinine (μmol/L, IQR)	65.00 (54.0–78.0)	65.00 (54.00–78.00)	70.00 (53.25–82.50)	0.126
BUN (mmol/L, IQR)	5.56 (4.48–6.92)	5.55 (4.47–6.92)	6.00 (4.98–7.49)	0.013
CK-MB (U/L, IQR)	29.40 (15.00–94.00)	29.30 (15.0–93.5)	34.70 (17.77–126.93)	0.112
CK (U/L, IQR)	257.00 (102.00–831.50)	257.00 (101.0–827.0)	249.00 (117.75–1440.50)	0.231
D-dimer (mg/L, IQR)	0.47 (0.27–0.87)	0.46 (0.27–0.86)	1.10 (0.62–2.28)	<0.001
AST (U/L, IQR)	45.00 (26.00–100.50)	44.00 (26.0–100.0)	58.00 (33.00–196.50)	0.012
ALT (U/L, IQR)	31.00 (21.00–48.00)	31.00 (21.0–48.0)	38.50 (26.50–67.00)	0.007
PT (s, IQR)	13.00 (11.80–13.80)	13.0 (11.80–13.80)	13.70 (12.93–14.67)	<0.001
Prothrombin activity (%, IQR)	98.00 (86.0–108.0)	98.0 (86.00–108.10)	87.10 (73.25–97.00)	<0.001
INR (IQR)	1.02 (0.97–1.09)	1.02 (0.96–1.09)	1.07 (1.02–1.17)	<0.001
TT (s, IQR)	17.20 (16.40–18.40)	17.20 (16.40–18.40)	16.75 (16.02–18.15)	0.036

IQR, interquartile range; ICT, intracardiac thrombosis; AMI, acute myocardial infarction; AWMI, anterior wall myocardial infarction; AF, atrial fibrillation; CKD, chronic kidney disease; Low HDLC, low high-density lipoprotein cholesterol; WBC, white blood cell; NEUs, neutrophils; LYMs, lymphocytes; BUN, blood urea nitrogen; CK-MB, creatine kinase-MB; CK, creatine kinase; AST, aspartate aminotransferase; ALT, alanine aminotransferase; PT, prothrombin time; INR, international normalized ratio; and TT, thrombin time.

**Table 2:** The results of the logistic regression analysis within the initial model.

	Univariate Regression		Multivariate Analysis	
Variables	OR (95% CI)	*p*-Value	OR (95% CI)	*p*-Value
Gender (male)	2.936 (1.101–6.024)	0.029	**2.936 (1.322–6.751)**	**0.009**
AWMI	5.761 (2.808–11.819)	<0.001	**5.186 (2.569–10.467)**	**<0.001**
Ventricular aneurysm	20.657 (11.779–36.226)	<0.001	**21.216 (12.455–36.183)**	**<0.001**
Killip I	1.534 (0.508–4.634)	0.448		
Killip II	2.667 (0.866–8.216)	0.087		
NEUs (×10^9^/L)	0.927 (0.853–1.007)	0.073		
LYMs (×10^9^/L)	0.852 (0.553–1.312)	0.467		
BUN (mmol/L)	1.069 (0.998–1.146)	0.058		
D-dimer (mg/L)	1.001 (0.974–1.029)	0.929		
AST (U/L)	1.000 (0.999–1.001)	0.683		
ALT (U/L)	1.001 (1.000–1.002)	0.199		
PT (s)	1.019 (0.998–1.040)	0.072		
Prothrombin activity (%)	1.039 (1.018–1.062)	<0.001	**1.039 (1.018–1.062)**	**<0.001**
INR	0.501 (0.131–1.911)	0.311		
TT (s, IQR)	1.003 (0.998–1.008)	0.207		

AWMI, anterior wall myocardial infarction. Variables of statistical significance are displayed in bold.

**Table 3:** The results of the logistic regression analysis in the sensitivity analysis.

	Univariate Regression		Multivariate Analysis	
Variables	OR (95% CI)	*p*-Value	OR (95% CI)	*p*-Value
Gender (male)	2.837 (1.037–7.079)	0.025	**2.564 (1.506–5.814)**	**0.005**
AWMI	13.052 (6.64–25.658)	<0.001	**4.578 (2.155–9.728)**	**<0.001**
Ventricular aneurysm	43.362 (25.925–72.525)	<0.001	**22.253 (12.239–40.459)**	**<0.001**
Killip I	0.378 (0.228–0.625)	<0.001	1.359 (0.459–4.022)	0.579
Killip II	2.650 (1.558–4.509)	<0.001	1.868 (0.615–5.671)	0.27
NEUs (×10^9^/L)	1.056 (0.992–1.123)	0.085		
LYMs (×10^9^/L)	0.658 (0.436–0.994)	0.047	0.969 (0.627–1.498)	0.887
BUN (mmol/L)	1.064 (1.004–1.042)	0.037	0.998 (0.972–1.025)	0.876
D-dimer (mg/L)	1.000 (1.004–1.042)	0.017	1.059 (0.978–1.147)	0.156
AST (U/L)	1.000 (1.000–1.001)	0.023	1.000 (0.999–1.001)	0.651
ALT (U/L)	1.001 (1.000–1.002)	0.134		
PT (s)	1.020 (0.998–1.043)	0.080		
Prothrombin activity (%)	1.026 (1.015–1.036)	<0.001	**1.025 (1.018–1.062)**	**<0.001**
INR	0.520 (0.998–1.575)	0.052		
TT (s, IQR)	1.004 (1.000–1.008)	0.062		

AWMI, anterior wall myocardial infarction. Variables of statistical significance are displayed in bold.

## Data Availability

Raw data cannot be provided due to laboratory policies and confidentiality agreements. However, we are available for any inquiries or further clarification if needed.

## Abbreviations

The following abbreviations are used in this manuscript:

IQR	Interquartile range
ICT	Intracardiac thrombosis
AMI	Acute myocardial infarction
AWMI	Anterior wall myocardial infarction
AF	Atrial fibrillation
CKD	Chronic kidney disease
HDLC	High-density lipoprotein cholesterol
WBC	White blood cell
NEUs	Neutrophils
LYMs	Lymphocytes
BUN	Blood urea nitrogen
CK-MB	Creatine kinase-MB
CK	Creatine kinase
NT-proBNP	N-terminal pro-B-type natriuretic peptide
AST	Aspartate aminotransferase
ALT	Alanine aminotransferase
PT	Prothrombin time
INR	international normalized ratio
TT	Thrombin time
ROC curve	Receiver operating characteristic curve
AUC	Area under the ROC curve

## References

[1] Anderson J.L., Morrow D.A. (2017). Acute Myocardial Infarction. N. Engl. J. Med..

[2] Gong F.F., Vaitenas I., Malaisrie S.C., Maganti K. (2021). Mechanical complications of acute myocardial infarction: A review. JAMA Cardiol..

[3] Byrne R.A., Rossello X., Coughlan J., Barbato E., Berry C., Chieffo A., Claeys M.J., Dan G.-A., Dweck M.R., Galbraith M. (2024). 2023 ESC Guidelines for the management of acute coronary syndromes: Developed by the task force on the management of acute coronary syndromes of the European Society of Cardiology (ESC). Eur. Heart J. Acute Cardiovasc. Care.

[4] Vaideeswar P., Divate S., Harke M. (2012). Intracardiac thrombi in extracardiac disorders: An autopsy study. Cardiovasc. Pathol..

[5] Wang P., Ye X., Yan D., Peng Y., Zhang Z. (2022). Incidence and risk factors of left ventricular thrombus in acute ST-segment elevation myocardial infarction treated by primary percutaneous coronary intervention: A meta-analysis. Med. Princ. Pract..

[6] Leow A.S., Sia C.H., Tan B.Y., Chan M.Y., Loh J.P. (2020). Characterisation of patients with acute myocardial infarction complicated by left ventricular thrombus. Eur. J. Intern. Med..

[7] Patel M., Wei X., Weigel K., Gertz Z.M., Kron J., Robinson A.A., Trankle C.R. (2021). Diagnosis and treatment of intracardiac thrombus. J. Cardiovasc. Pharmacol..

[8] McCarthy C.P., Vaduganathan M., McCarthy K.J., Januzzi J.L., Bhatt D.L., McEvoy J.W. (2018). Left ventricular thrombus after acute myocardial infarction: Screening, prevention, and treatment. JAMA Cardiol..

[9] Weinsaft J.W., Kim H.W., Shah D.J., Klem I., Crowley A.L., Brosnan R., James O.G., Patel M.R., Heitner J., Parker M. (2008). Detection of left ventricular thrombus by delayed-enhancement cardiovascular magnetic resonance prevalence and markers in patients with systolic dysfunction. J. Am. Coll. Cardiol..

[10] Chang P., Xiao J., Hu Z., Kwan A.C., Fan Z. (2022). Imaging of left heart intracardiac thrombus: Clinical needs, current imaging, and emerging cardiac magnetic resonance techniques. Ther. Adv. Cardiovasc. Dis..

[11] Boivin-Proulx L.-A., Ieroncig F., Demers S.-P., Nozza A., Soltani M., Ghersi I., Verreault-Julien L., Alansari Y., Massie C., Simard P. (2023). Contemporary incidence and predictors of left ventricular thrombus in patients with anterior acute myocardial infarction. Clin. Res. Cardiol..

[12] Levine G.N., Bates E.R., Bittl J.A., Brindis R.G., Fihn S.D., Fleisher L.A., Granger C.B., Lange R.A., Mack M.J., Mauri L. (2016). 2016 ACC/AHA guideline focused update on duration of dual antiplatelet therapy in patients with coronary artery disease: A report of the American College of Cardiology/American Heart Association Task Force on Clinical Practice Guidelines: An update of the 2011 ACCF/AHA/SCAI guideline for percutaneous coronary intervention, 2011 ACCF/AHA guideline for coronary artery bypass graft surgery, 2012 ACC/AHA/ACP/AATS/PCNA/SCAI/STS guideline for the diagnosis and management of patients with stable ischemic heart disease, 2013 ACCF/AHA guideline for the management of ST-elevation myocardial infarction, 2014 AHA/ACC guideline for the management of patients with non–ST-elevation acute coronary syndromes, and 2014 ACC/AHA guideline on perioperative cardiovascular evaluation and management of patients undergoing noncardiac surgery. Circulation.

[13] Bhatt D.L., Lopes R.D., Harrington R.A. (2022). Diagnosis and treatment of acute coronary syndromes: A review. JAMA.

[14] Bittl J.A., Baber U., Bradley S.M., Wijeysundera D.N. (2016). Duration of dual antiplatelet therapy: A systematic review for the 2016 ACC/AHA guideline focused update on duration of dual antiplatelet therapy in patients with coronary artery disease: A report of the American College of Cardiology/American Heart Association Task Force on Clinical Practice Guidelines. J. Am. Coll. Cardiol..

[15] Strom J., Manning W. (2017). Identification of Intracardiac Thrombus. Essential Echocardiography: A Companion to Braunwald’s Heart Disease.

[16] Shah T., Haimi I., Yang Y., Gaston S., Taoutel R., Mehta S., Lee H.J., Zambahari R., Baumbach A., Henry T.D. (2021). Meta-analysis of gender disparities in in-hospital care and outcomes in patients with ST-segment elevation myocardial infarction. Am. J. Cardiol..

[17] Medzikovic L., Azem T., Sun W., Rejali P., Esdin L., Rahman S., Dehghanitafti A., Aryan L., Eghbali M. (2023). Sex differences in therapies against myocardial ischemia-reperfusion injury: From basic science to clinical perspectives. Cells.

[18] Kaplan A., Abidi E., Diab R., Ghali R., Al-Awassi H., Booz G.W., Zouein F.A. (2022). Sex differences in cardiac remodeling post myocardial infarction with acute cigarette smoking. Biol. Sex Differ..

[19] D’Onofrio G., Safdar B., Lichtman J.H., Strait K.M., Dreyer R.P., Geda M., Spertus J.A., Krumholz H.M. (2015). Sex differences in reperfusion in young patients with ST-segment–elevation myocardial infarction: Results from the VIRGO study. Circulation.

[20] Díez-Delhoyo F., Gutiérrez-Ibañes E., Sanz-Ruiz R., Vázquez-Álvarez M.E., González Saldívar H., Rivera Juárez A., Sarnago F., Martínez-Sellés M., Bermejo J., Soriano J. (2019). Prevalence of microvascular and endothelial dysfunction in the nonculprit territory in patients with acute myocardial infarction: The FISIOIAM study. Circ. Cardiovasc. Interv..

[21] Grover-Páez F., Zavalza-Gómez A.B. (2009). Endothelial dysfunction and cardiovascular risk factors. Diabetes Res. Clin. Pract..

[22] Choi U.L., Park J.-H., Sun B.J., Oh J.K., Seong S.W., Lee J.-H., Choi S.W., Jeong J.-O., Kwon I.S., Seong I.-W. (2018). Impaired left ventricular diastolic function is related to the formation of left ventricular apical thrombus in patients with acute anterior myocardial infarction. Heart Vessel..

[23] Keren A., Goldberg S., Gottlieb S., Klein J., Schuger C., Medina A., Tzivoni D., Stern S. (1990). Natural history of left ventricular thrombi: Their appearance and resolution in the posthospitalization period of acute myocardial infarction. J. Am. Coll. Cardiol..

[24] Ram P., Shah M., Sirinvaravong N., Lo K.B., Patil S., Patel B., Tripathi B., Garg L., Figueredo V. (2018). Left ventricular thrombosis in acute anterior myocardial infarction: Evaluation of hospital mortality, thromboembolism, and bleeding. Clin. Cardiol..

[25] Delewi R., Zijlstra F., Piek J.J. (2012). Left ventricular thrombus formation after acute myocardial infarction. Heart.

[26] Camaj A., Fuster V., Giustino G., Bienstock S.W., Sternheim D., Mehran R., Dangas G.D., Kini A., Sharma S.K., Halperin J. (2022). Left ventricular thrombus following acute myocardial infarction: JACC state-of-the-art review. J. Am. Coll. Cardiol..

[27] Rathore S.S., Berger A.K., Weinfurt K.P., Schulman K.A., Oetgen W.J., Gersh B.J., Solomon A.J. (2000). Acute myocardial infarction complicated by atrial fibrillation in the elderly: Prevalence and outcomes. Circulation.

[28] Espe E.K., Aronsen J.M., Eriksen M., Sejersted O.M., Zhang L., Sjaastad I. (2017). Regional dysfunction after myocardial infarction in rats. Circ. Cardiovasc. Imaging.

[29] Leancă S.A., Crișu D., Petriș A.O., Afrăsânie I., Genes A., Costache A.D., Tesloianu D.N., Costache I.I. (2022). Left ventricular remodeling after myocardial infarction: From physiopathology to treatment. Life.

[30] Low C.J., Leow A.S.-T., Syn N.L.-X., Tan B.Y.-Q., Yeo L.L.-L., Tay E.L.-W., Yeo T.-C., Chan M.Y.-Y., Loh J.P.-Y., Sia C.-H. (2021). Outcomes of left ventricular thrombosis in post-acute myocardial infarction patients stratified by antithrombotic strategies: A meta-analysis with meta-regression. Int. J. Cardiol..

[31] Hansen C.H., Ritschel V., Halvorsen S., Andersen G., Bjørnerheim R., Eritsland J., Arnesen H., Seljeflot I. (2015). Markers of thrombin generation are associated with myocardial necrosis and left ventricular impairment in patients with ST-elevation myocardial infarction. Thromb. J..

[32] Zhang Q., Si D., Zhang Z., Wang C., Zheng H., Li S., Huang S., Zhang W. (2020). Value of the platelet-to-lymphocyte ratio in the prediction of left ventricular thrombus in anterior ST-elevation myocardial infarction with left ventricular dysfunction. BMC Cardiovasc. Disord..

[33] Smid M., Dielis A., Winkens M., Spronk H., Van Oerle R., Hamulyak K., Prins M., Rosing J., Waltenberger J., Ten Cate H. (2011). Thrombin generation in patients with a first acute myocardial infarction. J. Thromb. Haemost..

[34] Cirakoglu O.F., Aslan A.O., Yilmaz A.S., Şahin S., Akyüz A.R. (2020). Association between C-reactive protein to albumin ratio and left ventricular thrombus formation following acute anterior myocardial infarction. Angiology.

[35] Osherov A.B., Borovik-Raz M., Aronson D., Agmon Y., Kapeliovich M., Kerner A., Grenadier E., Hammerman H., Nikolsky E., Roguin A. (2009). Incidence of early left ventricular thrombus after acute anterior wall myocardial infarction in the primary coronary intervention era. Am. Heart J..

[36] Gianstefani S., Douiri A., Delithanasis I., Rogers T., Sen A., Kalra S., Charangwa L., Reiken J., Monaghan M., MacCarthy P. (2014). Incidence and predictors of early left ventricular thrombus after ST-elevation myocardial infarction in the contemporary era of primary percutaneous coronary intervention. Am. J. Cardiol..

